# Kramers–Kronig Diagnostic of Humidity-Induced Non-Idealities in Nanostructured Silica Capacitors

**DOI:** 10.3390/s26102957

**Published:** 2026-05-08

**Authors:** Bremnen Véliz, Sendey Vera, Sandra Bermejo, Albert Orpella, Manuel Domínguez-Pumar

**Affiliations:** 1 Electronics and Automation Department, Faculty of Systems and Telecommunications, Universidad Estatal Península de Santa Elena, La Libertad 240204, Ecuador; svera@upse.edu.ec; 2 MNT Group, Electronic Engineering Department, Universitat Politécnica de Catalunya, North Campus, 08034 Barcelona, Spain; sandra.bermejo@upc.edu (S.B.); albert.orpella@upc.edu (A.O.); manuel.dominguez@upc.edu (M.D.-P.)

**Keywords:** Kramers-Kronig, nanostructured silica, humidity dependence, metal-insulator-metal capacitor, electronic and ionic conduction

## Abstract

**Highlights:**

**What are the main findings?**

- Kramers–Kronig analysis demonstrates that high permittivity in nanostructured silica metal–insulator–metal capacitors is extrinsic, arising from humidity-induced deviations in linearity and causality.
- Augmented Transmission Line Modeling identifies moisture-driven ionic conduction and interfacial polarization as the specific mechanisms causing impedance consistency breakdown at relative humidity.

**What are the implications of the main findings?**

- Violations of Kramers–Kronig relations serve as diagnostic signatures of ionic transport mechanism rather than indicators of data reliability issues in porous dielectrics.
- The strong humidity dependence of Kramers–Kronig deviations reveals the onset of moisture-induced ionic conduction and interfacial polarization, with implications for the design of both humidity-sensitive devices and environmentally stable capacitors.

**Abstract:**

Metal–insulator–metal capacitors based on electrosprayed silica nanoparticles exhibit exceptionally high effective permittivity. However, their dielectric response is highly sensitive to ambient humidity, which can compromise data reliability. This study analyzes impedance characteristics of two silica nanoparticle-based MIM capacitors: (i) one measured under ambient conditions (0.1 Hz to 2 MHz) at 100/500 mV, and (ii) another under controlled relative humidity (RH) (40%, 70% and 90%) at 500 mV. Impedance consistency is rigorously assessed via Kramers–Kronig (KK) transforms. The first capacitor shows excellent KK consistency for real part Z′ (NRMSE = 3.3%), compatible with linear time-invariant assumptions. The second capacitor exhibits strong humidity-dependence deviations; NRMSE for Z′ rises from 14.5% at 40% RH to 141.2% at 90% RH, indicating linearity/causality breakdown from moisture-induced ionic conduction and interfacial polarization. These findings demonstrate that while increased humidity amplifies the effective dielectric response, it simultaneously introduces non-idealities that invalidate standard KK assumptions. Due to inherent microstructural variability between devices, humidity-dependent conclusions are derived from controlled intra-device analysis. Transmission Line Modeling confirms moisture enhances ionic network connectivity. Thus, KK analysis serves as a sensitive probe of environmental effects on nanostructured dielectrics, offering a framework to diagnose non-ideal behavior without a priori equivalent circuit models.

## 1. Introduction

Metal–insulator–metal (MIM) capacitors based on nanostructured dielectrics have attracted significant interest due to their potential for high capacitance density, and integration in modern microelectronic and sensing systems [1,2,3,4,5,6]. Among these, electrosprayed silica (SiO_2_) nanoparticle structures [7] have demonstrated exceptionally high effective permittivity—three orders of magnitude larger than that of bulk SiO_2_—making them potentially interesting for energy storage and environmental sensing applications. The electrosprayed porous shape layer, which encourages interfacial polarization and strong field confinement at the nanoscale, is responsible for this increased sensitivity. Similar structure–property relationships, where nanoscale morphology (particle size, packing density, and crystal structure) and interfacial effects govern functional behavior, have been reported in hybrid SiO_2_-based systems such as TiO_2_-SiO_2_ thin films [8].

However, environmental factors such as relative humidity and temperature have a significant impact on the electrical impedance spectroscopy [9] behavior of nanostructured silica capacitors [10]. The water molecules adsorbed into the nanopores can dissociate into mobile ions, introducing ionic conductivity that substantially alter the impedance response [11]. While the capacitive response and equivalent-circuit modeling of electrosprayed SiO_2_ structures have been previously reported for similar devices [12], the physical reliability of the impedance data—particularly under controlled humidity—and the underlying mechanisms governing the observed high permittivity remain unexplored.

Kramers–Kronig (KK) relations are frequently used to evaluate the physical validity of the measured impedance data. Without the need for an equivalent circuit model, the KK relations [13] offer a rigorous framework to verify whether experimental impedance data meets linearity, causality, and stability [14] requirements. In the absence of KK criteria, equivalent circuit models may yield misleading parameters or questionable impedance data. While practical implementations may require data extrapolation, sometimes assisted by equivalent circuit models, the core validation is based on the mutual consistency between the real and imaginary parts of the impedance [15].

This work addresses this gap by looking at Kramers–Kronig analysis, which serves not as a just pass/fail test, but as a diagnostic tool to reveal the extrinsic origins of the dielectric enhancement. Although Kramers–Kronig relations are often used as a consistency check in impedance spectroscopy, deviations from KK relations do not necessarily imply a problem of the quality of the experimental data. Instead, they may reveal hidden non-idealities such as time-dependent ionic transport, interfacial polarization, or environmental coupling, which are particularly relevant in nanostructured dielectric materials.

In this study, we investigate the impedance characteristics of two distinct electrosprayed SiO_2_ MIM capacitors: one was characterized in regular ambient laboratory conditions with ambient air, and another tested under specific controlled relative humidity levels (40%, 70%, and 90%). To apply the KK transform, we use the discretized formulation proposed by Orazem and Tribollet [16], which accounts for the high-frequency limit and keeps numerical stability. Our analysis demonstrates that while the ambient-condition device exhibits excellent KK consistency, the humidity-exposed capacitor shows increasing deviation with rising moisture content, which appears to be linked to ionic conduction plus interfacial polarization.

## 2. Materials and Methods

### 2.1. Fabrication of Electrosprayed SiO_2_ Nanostructures

The silica nanoparticles, dispersed in colloidal fluid consisting of 95% deionized water and 5% solid content, were purchased from microParticles GmbH, Berlin, Germany.

First, the bottom aluminum electrode was defined on a glass substrate by standard photolithography and thermal evaporation, followed by a lift-off process. The silica nanoparticles were then deposited by electrospray in a cone-jet regime. A stainless-steel dispensing needle with an inner diameter of 0.18 mm was placed at a distance of 6 cm from the bottom electrode. A high DC voltage (Ultravolt, New York, NY, United States) difference of approximately 7–8 kV was applied between the nozzle and the bottom plate electrode. The colloidal fluid was injected at a constant flow rate of 0.3 mL/h using an infusion pump (B.Braun, Melsungen, Germany). The deposition time was of approximately 10–12 min, after which the layer was dried for 30 min. Subsequently, the top aluminum electrode was deposited by thermal evaporation through a shadow mask.

Two metal–insulator–metal (MIM) capacitors were fabricated following this procedure, with active electrode areas of 3.24 mm^2^ and 15.9 mm^2^. Full fabrication details are provided [11].

### 2.2. Characterization

Impedance spectroscopy measurements were performed using a Hioki IM3590 (Tokio, Japan) (from 0.1 Hz to 100 kHz) and a 4294 A semiconductor analyzer (Agilent Technologies, Kobe, Japan) (from 100 kHz to 2 MHz). Two measurement protocols were as follows:

Capacitor 1: Characterized under ambient laboratory conditions (uncontrolled humidity), with the excitation voltage of 100 mV and 500 mV.

Capacitor 2: Measured inside a humidity-controlled chamber at fixed 500 mV (rms), with humidity precisely set to 40%, 70% and 90%, over 0.1 Hz to 100 kHz. Temperature was maintained at 25 °C.

### 2.3. Kramers–Kronig Validation

The physical consistency of the impedance data was evaluated using linear Kramers–Kronig (KK) transforms. Prior to Kramers–Kronig transformation [17], the measured impedance was converted to the passive sign convention, where a capacitive imaginary impedance is positive. The KK reconstruction was implemented using the discretized integral formulation using logarithmic frequency spacing over the measured frequency range (0.1 Hz to 100 kHz for Capacitor 2, and 0.1 Hz to 2 MHz for Capacitor 1), with no extrapolation to unmeasured frequencies. The singular point (i = j) is excluded by taking the Cauchy principal value, and the trapezoidal rule is applied in the log-frequency domain. This approach improves numerical stability and integration accuracy within the measured frequency window. The normalized root-mean-square error (NRMSE) were used as metrics to quantify deviation between measured and KK-reconstructed impedance.

Although the Kramers–Kronig relations formally require data over an infinite frequency range, the measured impedance spans six decades (0.1 Hz to 100 kHz), which is sufficient to assess relative consistency and identify humidity-induced deviations from ideal behavior within the frequency window of interest.

### 2.4. Formulation

The complex impedance Z over a given frequency range is expressed in terms of their real and imaginary parts, such as

(1) $$Z=Z^{\prime }+jZ^{\prime \prime }$$

The impedance can be expressed in terms of a complex permittivity, $\varepsilon =\varepsilon^{\prime }+j\varepsilon^{\prime \prime }$, as

(2) $$Z=\frac{1}{j\omega C}$$

where C is the complex capacitance:

(3) $$C=\frac{A\left(\varepsilon^{\prime }+j\varepsilon^{\prime \prime }\right)}{t}$$

The relative (effective) dielectric permittivity is

(4) $${\varepsilon^{\prime }}_{r}=\frac{\varepsilon^{\prime }}{\varepsilon_{O}}$$

where ε′ is the real permittivity, *ε_O_* as the permittivity of free space.

The real permittivity can then be expressed as

(5) $$\varepsilon^{\prime }=-\frac{t}{A}\frac{Z^{\prime \prime }}{{\omega \left|Z\right|}^{2}}$$

where ω is angular frequency, *A* is electrode area, *t* is film thickness, and |*Z*| is magnitude of the complex impedance.

The imaginary part of the impedance can be expressed as

(6) $$Z^{\prime \prime }=-\frac{t}{A\omega }\left(\frac{\varepsilon^{\prime }}{{\varepsilon^{\prime }}^{2}+{\varepsilon^{\prime \prime }}^{2}}\right)$$

The loss tangent is a dimensionless metric that quantifies the fraction of the stored electrical energy dissipated as heat per AC cycle. It is defined as the ratio of the imaginary to the real parts of the complex permittivity:

(7) $$\tan\delta =\frac{\varepsilon^{\prime \prime }}{\varepsilon^{\prime }}=\frac{Z^{\prime }}{\left|Z^{\prime \prime }\right|}$$

The Kramers–Kronig (K–K) relations is used to verify the validity and consistency of the measured impedance data with the fundamental requirements of a linear, causal, and time-invariant system.

(8) $${Z^{\prime }}_{kk}=Z^{\prime }\left(\infty \right)+\frac{2}{\pi }\int_{0}^{\infty }\frac{xZ^{\prime \prime }\left(x\right)-\omega Z^{\prime \prime }\left(\omega \right)}{x^{2}-\omega^{2}}dx$$

(9) $${Z^{\prime \prime }}_{kk}=-\frac{2}{\pi }\int_{0}^{\infty }\frac{\omega Z^{\prime }\left(x\right)-\omega Z^{\prime }\left(\omega \right)}{x^{2}-\omega^{2}}dx$$

where *Z*′(∞) represents the series resistance of the device.

To discretize, the variable is changed to a logarithmic scale:

(10) $$u=ln\left(x\right)\;\to du=\frac{dx}{x}$$

Evaluating at zero, the value of the new variable is

(11) $$u\left(0\right)=ln\left(0\right)=-\infty$$

At the infinite limit:

(12) $$u\left(\infty \right)=ln\left(\infty \right)=\infty$$

The integrals can be written as

(13) $${Z^{\prime }}_{kk}=\frac{2}{\pi }\int_{-\infty }^{\infty }\frac{xZ^{\prime \prime }\left(x\right)-\omega Z^{\prime \prime }\left(\omega \right)}{{x^{2}-\omega }^{2}}xdu$$

(14) $${Z^{\prime \prime }}_{kk}=-\frac{2}{\pi }\int_{-\infty }^{\infty }\frac{\omega Z^{\prime }\left(x\right)-\omega Z^{\prime }\left(\omega \right)}{{x^{2}-\omega }^{2}}xdu$$

By discretizing the integral, the differential du becomes an increment *Δu*:

(15) $$du=∆u=∆\left(\ln\left(x\right)\right)$$

Given *ω_j_* = x, *ω_i_ = ω*, and using (13), the integrals can be approximated as a sum using the rectangle/trapezium rule in *u*:

(16) $${Z^{\prime }}_{kk}=\frac{2}{\pi }\sum_{j\neq i}\frac{{\omega_{j}}^{2}Z^{\prime \prime }\left(\omega_{j}\right)-\omega_{j}\omega_{i}Z^{\prime \prime }\left(\omega_{i}\right)}{{\omega_{j}}^{2}-{\omega_{i}}^{2}}∆\left[ln{(\omega }_{j})\right]$$

(17) $${Z^{\prime \prime }}_{kk}=-\frac{2}{\pi }\sum_{j\neq i}\frac{\omega_{i}\omega_{j}Z^{\prime }\left(\omega_{j}\right)-\omega_{i}\omega_{j}Z^{\prime }\left(\omega_{i}\right)}{{\omega_{j}}^{2}-{\omega_{i}}^{2}}∆\left[ln{(\omega }_{j})\right]$$

The value j = i is excluded because near w = ω the integrand becomes infinite. In electrical impedance spectroscopy, frequency often varies over many decades, so logarithmic spacing is convenient because it gives the same number of points per decade of frequency, and it also improves the numerical stability of the Kramers–Kronig formulas because the summation terms are more balanced.

The residuals [14] are defined as

(18) $$∆Z^{\prime }=\frac{\left|Z^{\prime }-{Z^{\prime }}_{rr}\right|}{\left|Z\right|}$$

(19) $$∆Z^{\prime \prime }=\frac{\left|Z^{\prime \prime }-{Z^{\prime \prime }}_{rr}\right|}{\left|Z\right|}$$

Normalized root-mean-square errors are normalized with respect to the value module measured at that point, and are defined as

(20) $$NRMS{E^{\prime }}_{Z}=100\%\sqrt{\frac{1}{N}\sum_{n}{\left(\frac{Z^{\prime }\left(n\right)-{Z^{\prime }}_{rr}(n)}{\left|Z\left(n\right)\right|}\right)}^{2}}$$

(21) $$NRMS{E^{\prime \prime }}_{Z}=100\%\sqrt{\frac{1}{N}\sum_{n}{\left(\frac{Z^{\prime \prime }\left(n\right)-{Z^{\prime \prime }}_{rr}(n)}{\left|Z\left(n\right)\right|}\right)}^{2}}$$

### 2.5. Transmission Line Modeling (TLM)

**Figure 1:**
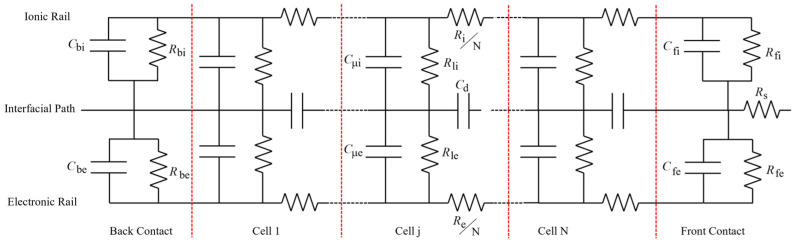
Augmented hybrid Transmission Line Model (TLM) for nanostructured silica MIM capacitor under high humidity. Two parallel rails represent ionic and electronic conduction, coupled by chemical capacitors. Leakage resistances (R_l_) model shunt paths through condensed water bridges.

To accurately capture the frequency response under high humidity, an augmented hybrid Transmission Line Model (TLM) was employed, as illustrated in Figure 1. This configuration divides the nanoparticle network into a bulk region and an interface region, each comprising discrete cells. Based on the impedance formalism for mixed ionic–electronic conductors (MIECs) [18], the bulk regions are characterized by distributed resistances (r_i_, r_e_) and chemical capacitances (C_μi_, C_μe_), while terminal impedances at the back and front contacts (C_bi_, R_bi_, C_be_, R_be_, C_fi_, R_fi_, C_fe_, R_fe_) account for interfacial charge transfer and reaction processes coupled at metal/water and water/SiO_2_ interfaces. The geometrical dielectric capacitance is represented by C_d_. A key feature of this augmented model is the inclusion of parallel leakage resistances, R_li_ and R_le_, across the ionic and electronic branches. These parameters account for shunt conduction paths, likely through condensed water bridges that bypass the structured surface transport, providing a physical basis for the observed low-frequency impedance roll-off at high RH levels.

## 3. Results

### 3.1. SEM Characterization

**Figure 2:**
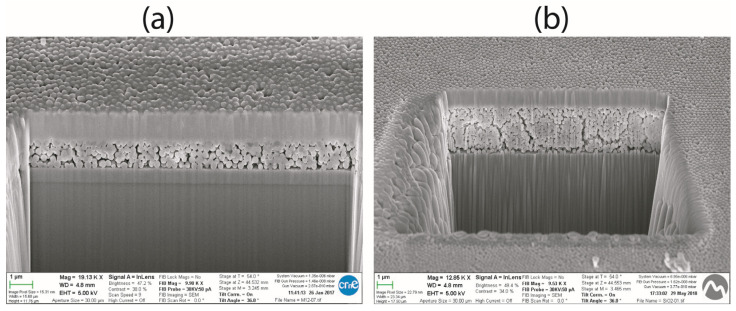
Drilled cross-section SEM images of (**a**) First Electrosprayed SiO_2_ nanostructure, (**b**) Second Electrosprayed SiO_2_ nanostructure.

Figure 2 reports the cross-sectional SEM images of the fabricated devices with SiO_2_ nanoparticles. The cross-sectional images were obtained after focused ion beam (FIB) milling, a destructive preparation technique that provides high-quality imaging of the internal layer structure but renders the devices unsuitable for further electrical testing. The cavities visible in Figure 2a,b correspond to trenches intentionally milled by the FIB to expose the internal stack, enabling direct measurement of the thickness and morphology of the nanoparticle layers in cross-section.

Figure 2a reports the cross-sectional SEM images of SiO_2_ nanoparticles Capacitor 1. The 255 nm silica nanospheres are randomly close-packed, forming a network with interparticle voids (pores) of approximately 20–40 nm. The nanoparticle diameter was estimated by direct measurements on the SEM images using ImageJ 1.54g, yielding an average value of 250.7 nm, in good agreement with the nominal 255 nm specified by the manufacturer. The total thickness of the electrosprayed nanoparticle dielectric layer is 1.21 μm; meanwhile, thicknesses of 0.3 μm and 1.0 μm are the bottom and top layer thicknesses respectively.

Figure 2b corresponds to Capacitor 2, which has a thicker nanoparticle layer of 3.11 μm and similar irregular upper surface. The measured average nanoparticle diameter is 252.4 nm, confirming the consistency of particle size across samples. The nanoparticles form a high surface area network of interconnected pores that enable water vapor condensation and capillary adsorption. The larger thickness observed in Capacitor 2 is attributed to longer electrospray deposition time and slightly different flow conditions. The bottom and top metal layer are 0.6 μm and 1.2 μm thick, respectively.

Quantitative image analysis was performed using ImageJ (Fiji) to estimate the two-dimensional porosity (area fraction of interparticle voids) from the SEM cross-sections. For Capacitor 1 (Figure 2a), the porosity was 16.2%, and for Capacitor 2 (Figure 2b), it was 20.0%. These values are consistent with a dense random packing of monodisperse spheres, where the volumetric porosity is known to be approximately 36% [19]. The lower porosity observed in 2D sections is attributed to stereological effects and the limited contrast resolution of SEM imaging, which may underestimate sub-resolution interparticle voids [20].

It should be noted that this analysis is based on a single cross-section per device and therefore provides a localized description of the microstructure. As a result, the reported values should be interpreted as qualitative indicators rather than statistically representative of the entire film or absolute metrics.

The observed morphology is consistent with previous reports on electrosprayed silica nanoparticle films and on monodispersed SiO_2_ nanospheres deposited by drop-casting [10]. Therefore, the structural features of our devices are not claimed as novel. Instead, the novelty of the present work lies in the application of Kramers–Kronig analysis as a diagnostic tool to reveal humidity-induced non-linearities and causality violations in such nanostructured dielectrics.

### 3.2. Capacitor 1: Dielectric Characteristics at Room Humidity

**Figure 3:**
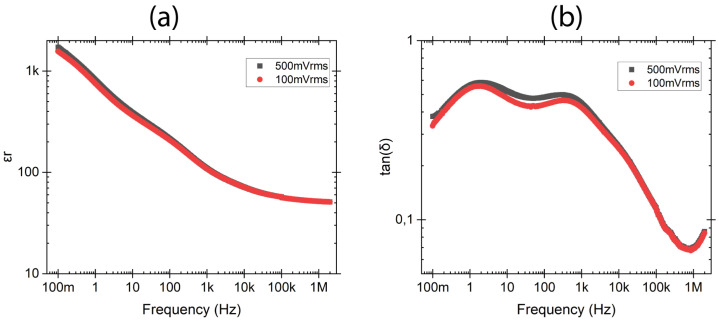
(**a**) Relative permittivity and (**b**) loss tangent for silica nanoparticles metal–insulator–metal capacitor.

As shown in Figure 3a, the silica capacitor exhibits a strong frequency-dependent dispersion of the relative permittivity (ε_r_′). The log-log plot reveals exceptionally high low-frequency permittivity values, reaching approximately 1726 (at 500 mV) and 1568 (at 100 mV). When the frequency increases, the permittivity drops rapidly to 109 at 1 kHz. Above approximately 1 kHz, the permittivity becomes independent of the excitation voltage and stabilizes at 51.1 at 1 MHz. This stabilized value is 13.1 times higher than that of bulk silica (~3.9), while at the lowest frequency the effective permittivity is 442.6 times higher.

Figure 3b shows the frequency dispersion of the loss tangent (tanδ) measured under 500 mV and 100 mV excitation. The loss tangent exhibits high values at low frequencies, reaching a maximum of approximately 0.58 at 2 Hz for 500 mVrms. As frequency increases, tanδ decreases steadily to a minimum of 0.067 at 0.85 MHz (for 100 mVrms), without a clear plateau up to 1 MHz. A similar decrease was observed in an Al/SiO_2_/p-Si capacitor from 50 kHz to 1 MHz [21]. A small voltage dependence is observed across the low-to-mid-frequency range (approximately 0.1 Hz to 2 kHz), where a higher applied voltage results in a greater loss tangent.

The slight increase in permittivity under 500 mV compared to 100 mV suggests a minor field-enhancement of interfacial polarization processes at low frequencies. The extremely high permittivity values are attributed to the cumulative effect of polarization at the electrode–dielectric interface, the interfaces between nanoparticles, and the presence of adsorbed moisture (which introduces mobile charge carriers), as noted in previous works. This response is therefore dominated by extrinsic interfacial and space-charge effects rather than intrinsic dielectric polarization.

The observed loss tangent behavior suggests the presence of field-assisted, nonlinear charge transport [22] in addition to interfacial polarization. The elevated losses at low frequencies are characteristic of conductive losses dominated by leakage current [23] and interfacial polarization in porous silica nanostructures [24]. Consequently, the capacitor is significantly more efficient in terms of stored-to-dissipated energy at high frequencies, where tanδ is reduced. This trade-off highlights that the high apparent permittivity at low frequencies is accompanied by significant energy dissipation and therefore does not represent a high intrinsic dielectric energy storage capability of the silica material.

### 3.3. Capacitor 1: Impedance Consistency with Kramers–Kronig Integrals

**Figure 4:**
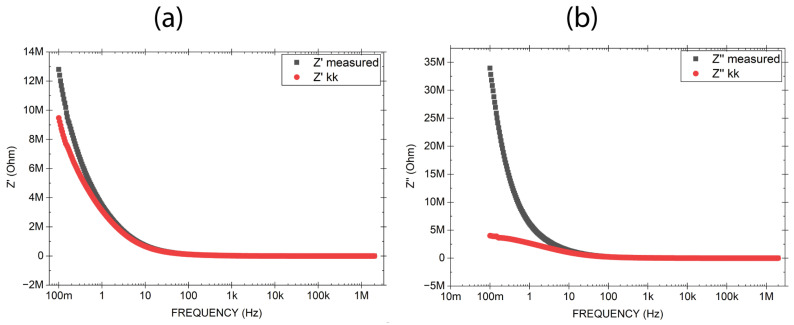
Spectrum comparison between the measured real (**a**) and imaginary part (**b**) of the impedance with the Kramers–Kronig (KK) impedances for an applied voltage of 500 mVrms: The Z”KK curve does not follow the Z” measured curve at low frequencies.

The physical consistency of the impedance data was evaluated using Kramers–Kronig (KK) transforms according to the formalism proposed by [16]. As shown in Figure 4a, the KK reconstruction yields excellent agreement for the real part of the impedance, with only a small deviation at low frequencies. The average residual is 2.13%, and the normalized root-mean-square error (NRMSE) is 3.3%. In contrast, the imaginary part exhibits a large deviation (Figure 4b), with an average residual of 18.13% and an NRMSE of 28.98%. Notably, when the KK integral is restricted to the high-frequency range (100 Hz–2 MHz), the NRMSE for Z″ drops to 4.5%.

The low NRMSE values for Z′ confirm that the resistive component satisfies the fundamental requirements of linearity, causality, and stability across the entire frequency range. The larger errors in Z″ are not indicative of poor data quality; rather, they reflect non-ideal physical processes inherent to the porous and moisture-absorbing nature of the SiO_2_ nanostructure, such as interfacial polarization and moisture-induced ionic conduction. These phenomena violate the strict Kramers–Kronig assumptions, especially at low frequencies. The marked improvement in Z″ consistency at high frequencies (NRMSE = 4.5%) confirms that the device behaves more linearly and causally when polarization and conduction transients have insufficient time to develop. The high fidelity of the real-part validation reinforces the reliability of the measured impedance for dielectric analysis. It suggests that at high frequencies, the capacitor behavior is governed primarily by the intrinsic dielectric properties of silica, while the discrepancies in the imaginary part at low frequencies faithfully capture the complex microstructural and environmental interactions of the material, rather than instrumental artifacts. It is important to note that while Capacitor 1 demonstrates that KK consistency is achievable in these nanostructures under ambient conditions, its NRMSE values serve primarily as a qualitative baseline, not as a direct numerical comparison with Capacitor 2, due to structural differences (electrode area, thickness, and microstructure).

### 3.4. Capacitor 2: Dielectric Characteristic at Controlled Humidity

It is important to emphasize that Capacitor 2 differs from Capacitor 1 not only in electrode area and dielectric thickness, but also in the stochastic microstructure of the electrosprayed silica nanoparticle assemblies. Although relative permittivity and loss tangent are, in principle, independent of device geometry, the effective dielectric characteristics may be strongly influenced by factors, such as pore connectivity and interfacial area, which vary from device to device. Therefore, the present analysis is focused how controlled relative humidity systematically affects the dielectric response within a given device.

**Figure 5:**
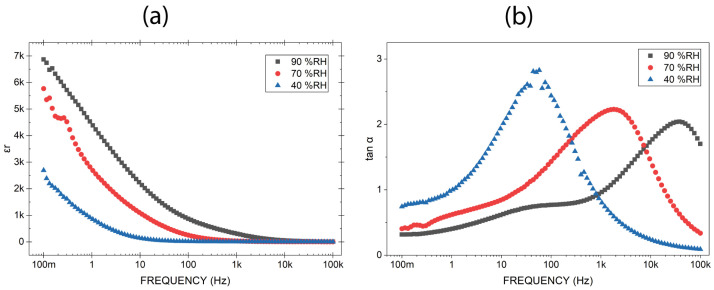
Effective permittivity ε′_r_ (**a**) and loss tanδ (**b**) as a function of frequency measured at 90% RH, 70% RH and 40% RH.

Figure 5a shows that increasing relative humidity leads to a marked enhancement of the effective permittivity. At 0.1 Hz and 90% RH, the relative permittivity reaches a maximum value of 6870, approximately 1761 times higher than the intrinsic permittivity of bulk silica (~3.9), while at 0.1 Hz and 40% RH, the effective permittivity is 2690. The extrinsic permittivity vanishes at high frequencies, where the response converges toward the intrinsic dielectric behavior.

Figure 5b reveals that the loss tangent decreases with increasing relative humidity in the low-frequency range (below 260 Hz). At 40% RH, tanδ crosses unity at low frequencies (around 1 Hz) and again at mid-frequencies (around 700 Hz). As humidity increases to 70% and 90% RH, both crossover frequencies shift to progressively higher values, for instance, at 90% RH, the first crossover shifts further to 1.2 kHz. At high frequencies (above 10 kHz), tanδ increases for all humidity levels.

The increase in effective permittivity with humidity confirms that water molecules enter the nanostructure and provide ionic carriers (e.g., H^+^ and OH^−^) that enhance interfacial polarization and increase the charge storage capacity. The decrease in tanδ at low frequencies reflects a relative enhancement in energy storage over dissipation, because the real permittivity (ε′) increases more rapidly than the imaginary permittivity (ε″), rather than a reduction in dissipative processes. Consequently, with increasing humidity, both the lower and upper frequency bounds where tanδ < 1 shift to higher frequencies, meaning that the capacitive-dominated region (tanδ < 1) moves toward higher frequencies. At high frequencies (above 10 kHz), tanδ increases because ε′ diminishes as interfacial polarization can no longer follow the rapidly alternating field.

### 3.5. Capacitor 2: Effect of Relative Humidity on the Kramers–Kronig Validation of Impedance Spectra for an Electrosprayed Silica Nanostructure

It should be noted that two MIM capacitors analyzed in this study differ in both electrodes area and dielectric thickness, resulting in distinct absolute impedance values. Therefore, the following analysis focuses on the qualitative trends associated by humidity, rather than direct quantitative comparison between devices. All conclusions regarding humidity-induced KK behavior are based exclusively on intra-device trends with Capacitor 2. Capacitor 1 serves as an illustrative reference to demonstrate that KK consistency is achievable in electrosprayed silica nanostructures under ambient conditions. While SEM-based image analysis provides a useful qualitative description of nanoparticle size and porosity, it does not constitute a statistically representative or fully controlled characterization of the microstructure. Therefore, device-to-device variability cannot be rigorously quantified, and inter-device comparisons remain illustrative rather than conclusive.

**Figure 6:**
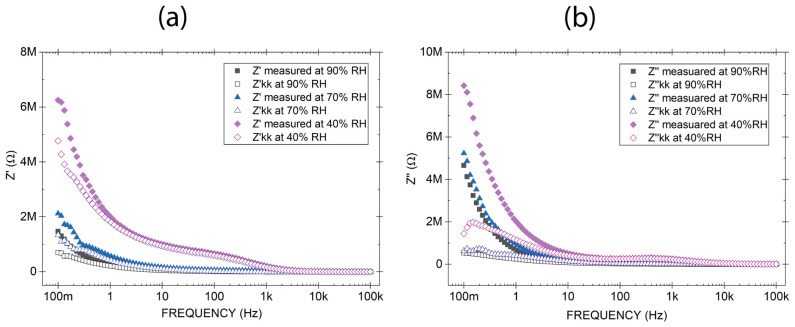
Comparison between measured and Kramers–Kronig reconstructed real (**a**) and imaginary (**b**) parts of impedance for an electrosprayed silica MIM capacitor under controlled relative humidity conditions (90% RH, 70% RH, and 40% RH). It demonstrates that increasing humidity progressively destroys KK consistency at low frequencies, indicating the emergence of slow ionic and interfacial processes incompatible with linear time-invariant assumptions.

The real part of the impedance under controlled relative humidity is shown in Figure 6a. As humidity increases, the real part of the impedance decreases and deviates from the impedance obtained by Kramers–Kronig transforms at low frequencies. The normalized root-mean-square (NRMSE) for Z′ was 141.2%, 94.5% and 14.5% at 90% RH, 70% RH and 40% RH respectively. This increasing divergence, particularly at elevated humidity levels and in the low-frequency data, suggests a failure to meet the conditions of linearity and causality required for KK validity.

In Figure 6a, the reduction in Z′ with increasing humidity is attributed to the absorption of water humidity into the porous electrospray silica layer, indicating that the moisture induced introduces time-dependent effects that prevent strict Kramers–Kronig consistency, particularly in the low-frequency range. This absorption phenomena enhances ionic conductivity across the dielectric, effectively lowering the overall resistive component of the impedance. In other words, moisture transforms the insulating silica nanostructure into a partially conductive medium, thereby reducing the real part of the impedance.

The imaginary component of the impedance, Z″, exhibits values decreasing as humidity increases (Figure 6b). At 1 Hz, for instance, Z″ is approximately 658 kΩ at 90% RH, 899 kΩ at 70% RH, and 1.9 MΩ at 40% RH. This trend, where higher humidity leads to lower Z″, is consistent with the strong increase in effective permittivity (ε′) due to interfacial polarization enhanced by water adsorption in the porous silica structure. Since Z″ is proportional to ε′ and inversely proportional to (ε′)^2^ + (ε″)^2^, as humidity increases, both ε′ and ε″ rise due to interfacial polarization and ionic conduction, but ε′ grows more than ε″, causing Z″ to decrease. Thus, the reduction in Z″ reflects the dominance of enhanced charge storage (high ε′) over dissipative processes, consistent with the observed decrease in tanδ (Figure 4b), which indicates improved energy storage efficiency despite the presence of ionic conduction.

The Kramers–Kronig validation further supports this interpretation: the normalized RMS error for Z″ reaches 51.6% at 90% RH, indicating significant deviation from linearity and causality due to non-ideal effects such as ionic conduction and interfacial polarization. In contrast, at 40% RH, the NRMS error drops to 27.1%, reflecting a more “ideal” capacitive behavior.

### 3.6. Capacitor 2: Physical Interpretation via TLM Fitting

**Figure 7:**
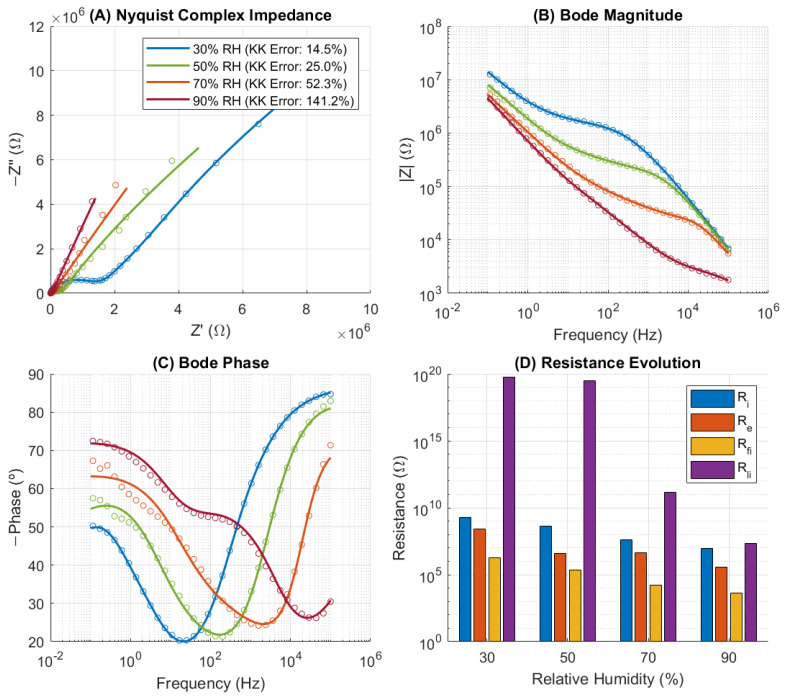
Transmission Line Model (TLM) fitting results for the nanostructured silica MIM capacitor under controlled relative humidity (30–90% RH). (**A**–**C**) Nyquist and Bode plots showing experimental impedance data (symbols) and TLM fits (solid lines) at representative humidity levels. (**D**) Evolution of key fitted parameters.

As illustrated in Figure 7, the TLM accurately captures the Nyquist and Bode responses across different relative humidity levels. The evolution of the fitted parameters (Figure 7D) shows a great shift. The characteristic resistances of the network: total ionic and electronic resistance (Ri, Re), leakage ionic resistance (Rli) and ionic front contact resistance (Rfi), exhibit a synchronized decrease of approximately three orders of magnitude. This coordinated reduction suggests that moisture does not only increase the number of charge carriers but fundamentally enhances the global connectivity of the ionic network through the percolation of adsorbed water layers. The complete set of fitted parameters is provided in Supplementary Table S1. While some terminal elements exhibited extreme values indicative of overparameterization in those specific branches (commons in distributed element models with multiple interfacial processes), bulk and leakage resistances show robust convergence.

The robustness of the TLM fitting was validated through starting-value dependence tests, ensuring that the converged core parameters reflect a stable global minimum rather than numerical artifacts. Furthermore, the convergence of back-contact resistances (Rbi and Rbe) to near-zero values across all RH conditions physically confirms their role as ideal ohmic interfaces. While the sensitivity to certain terminal polarization branches decreases at high humidity due to the strong shunting effect of the condensed moisture pathways, a single, unreduced global TLM topology was maintained across the entire humidity range. This approach prevents structural overparameterization bias while preserving the physical comparability of the extracted transport metrics.

## 4. Discussion

The exceptionally high effective permittivity observed at low frequencies (1726 for Capacitor 1; 2690 for Capacitor 2 at 40% RH) is a macroscopic response controlled by interfacial polarization [25]. This phenomenon, often referred to as Maxwell–Wagner–Sillars (MWS) polarization in composite systems, arises from accumulation of free charge at internal interfaces or phase boundaries with contrasting electrical conductivities. In our case, it physically originates from space-charge accumulation within the nanoporous silica network and at the electrode interface, and moisture-assisted ionic transport within nanoporous electrosprayed structure, rather than an inherent characteristic of SiO_2_. It is evident that slow polarization mechanisms predominate in the low-frequency zone due to the fast collapse of ε_r_′ with frequency (ε_r_′ = 51.1 at 1 MHz for Capacitor 1) and the substantial dependency on humidity and excitation voltage. While absolute permittivity values between Capacitor 1 and Capacitor 2 are influenced by device–device microstructural variability and should not be directly compared, the humidity-dependence enhancement observed within Capacitor 2 (2690 at 40% RH → 6870 at 90% RH) demonstrates the intra-device trend that is methodologically robust.

The frequency-dependent high permittivity observed in the present work is consistent with previous reports on nanostructured silica dielectrics, notably, the study by Sakthisabarimoorthi et al. [10], where large dielectric constants (e.g., 2368 at 1 Hz and 40 °C, dropping to ε_r_′ = 68 at 1 MHz) were attributed to interfacial polarization and charge accumulation within porous SiO_2_ networks. Similar to their observations, the enhancement is confined to the low-frequency regime and rapidly diminishes at higher frequencies, indicating the dominance of slow polarization mechanisms. Despite the strong dependence of permittivity on effective microstructure, a close agreement is observed with the dielectric response reported by Sakthisabarimoorthi. for silica nanostructures fabricated using a different processing route, providing independent and robust validation that the observed high permittivity is an extrinsic artifact, overwhelmingly dominated by interfacial polarization rather than an intrinsic property of silica.

The reduction in tanδ with humidity at low frequencies (Figure 4b) is not indicative of lower absolute dissipation, but rather of a relative increase in energy storage capacity (ε′) compared to losses (ε″). Thus, while the system becomes more efficient in terms of the ratio of stored to dissipated energy, the absolute energy loss remains significant. Although moisture causes the presence of a larger population of mobile ionic species, and therefore an increase in imaginary component ε″ associated with conductive losses, in the low-frequency regime, ionic motion remains partially blocked, contributing predominantly to energy storage rather than dissipation. At higher frequencies (above 10 kHz), this balance reverses, and the interfacial polarization can no longer follow the rapid alternating, causing ε′ to drop, thereby increasing tanδ.

This crossover behavior contrasts with the temperature-dependent trends reported by Sakthisabarimoorthi. While Sakthisabarimoorthi observed a reduction in tanδ with increasing temperature in a dry environment, our study examines that the effect of increasing relative humidity at constant temperature is a decrease in tanδ. In their case, thermal activation enhances charge mobility without introducing additional interfacial water layers, whereas in our system, humidity promotes the formation of hydrated interfaces that improved the interfacial polarization. The apparent discrepancy thus reflects distinct dominant mechanisms under humidity-controlled versus thermally driven conditions, rather than a contradiction, and further supports the interpretation that the exceptionally high permittivity in nanostructured silica is governed by extrinsic interfacial effects strongly modulated by environmental factors.

The Kramers–Kronig analysis reveals a fundamental distinction between measurements performed under ambient conditions and those under controlled humidity. While the ambient-condition capacitor shows excellent KK consistency for Z′, increasing relative humidity progressively destroys KK validity at low frequencies. This behavior does not indicate experimental artifacts, but rather the emergence of time-dependent and history-dependent processes, such as ionic drift and electrode polarization, which violate the assumptions of linear time-invariant systems.

We acknowledge that the Kramers–Kronig relations formally require integration from 0 to ∞. To test the sensitivity of our results to extrapolation, we performed constant extrapolation of Z′ and Z″ below 0.1 Hz (using the values at 0.1 Hz) and above 100 kHz/2 MHz (using the values at the highest measured frequency). This constant extrapolation, while mathematically simple, is physically unrealistic for a porous dielectric where Z′ and Z″ do not remain constant as frequency approaches DC. The extrapolated NRMSE values not only reduced the sensitivity to humidity but, in some cases, inverted the expected trend (higher humidity yielding lower NRMSE). Therefore, we adopt a conservative approach: the integrals are truncated at the measured frequency limits, and the resulting NRMSE values are interpreted as an empirical index of relative consistency within the measured window. This approach preserves the clear physical trend of increasing deviation with rising humidity, consistent with the emergence of moisture-induced ionic conduction and interfacial polarization. We emphasize that extending the measurable frequency range is preferable to simple constant extrapolation, which introduces more artifacts than it resolves.

A potential point of clarification should be addressed: why does tanδ decrease with humidity at low frequencies (Figure 5b) while KK deviations increase (Figure 6)? The answer lies in the different physical information captured by each metric. The loss tangent tanδ = ε″/ε′ measures the relative energy dissipation per cycle under the assumption of a linear, time-invariant system. As humidity increases, both ε′ and ε″ grow due to enhanced ionic conduction and Maxwell–Wagner–Sillars polarization. However, ε′ grows faster than ε″ in the low-frequency range, causing tanδ to decrease. In contrast, the KK deviation (NRMSE) does not measure the magnitude of losses but rather the failure of the data to satisfy the fundamental conditions of causality and linearity. The same ionic transport processes that boost ε′ also introduce time-dependent and nonlinear behavior (e.g., slow relaxation, history-dependent conduction), which directly violates the KK assumptions. Consequently, a decrease in tanδ does not imply a more ‘ideal’ or KK-compliant system; it merely reflects the relative enhancement of storage over dissipation within a system that is progressively less linear and causal. This distinction is critical for interpreting impedance data in humid porous dielectrics.

The breakdown of Kramers–Kronig (KK) consistency at high RH (NRMSE > 100%) correlates with the abrupt activation of leakage paths. At 90% RH, Rli falls significantly (to 2.3 × 10^+7^ Ω), becoming a dominant conduction mechanism. This suggests that the ‘High Permittivity’ measured at low frequencies is heavily influenced by a quasi-DC ionic shunt. Since KK transforms assume a linear, causal, and stationary dielectric response, the emergence of these humidity-induced conduction channels and interfacial charge accumulation introduces non-linearities that invalidate the KK assumptions, confirming that the massive increase in effective capacitance is coupled with a fundamental change in the transport regime.

Our previous work established a modified Randles circuit for these silica MIM capacitors and identified Poole–Frenkel emission and charge trapping as the dominant conduction mechanisms. Those studies already pointed to extrinsic, humidity-sensitive behavior, but relied on equivalent-circuit fitting. The present KK analysis provides a model-independent validation that the high permittivity is extrinsic and that humidity systematically destroys linearity and causality. Moreover, the Transmission Line Modeling introduced here captures, for the first time, the humidity-dependent evolution of ionic and electronic transport in these devices, extending our previous electrical characterization.

Specifically, the TLM analysis (Section 3.6) revealed a synchronized three-order-of-magnitude reduction in network resistance at high humidity, confirming the activation of moisture-induced ionic pathways. Thus, while the intrinsic porosity of the silica nanoparticle layers is a necessary condition for humidity sensitivity, the macroscopic dielectric response and its compliance with KK relations are governed by more complex interplay of layer thickness, pore connectivity, and interfacial quality.

The quantitative porosity analysis reveals that both devices have similar packing densities, with Capacitor 2 showing a slightly higher porosity (20.0%) compared to Capacitor 1 (16.2%). While these values are within the expected range for dense random packings of monodisperse spheres, they alone do not explain the markedly different humidity-dependent behavior observed in Capacitor 2. Instead, the stronger degradation of KK consistency in Capacitor 2 (NRMSE for Z′ rising from 14.5% at 40% RH to 141.2% at 90% RH) is attributed to a combination of factors: its greater layer thickness (3.11 μm vs. 1.21 μm), which provides a longer pathway for ionic conduction, and potentially a higher degree of pore connectivity, which facilitates the formation of percolating water networks.

From an application standpoint, these findings highlight a fundamental trade-off. While humidity dramatically enhances apparent permittivity, it simultaneously introduces non-ideal effects that compromise physical consistency and long-term stability. Therefore, electrosprayed silica nanostructures may be of interest for humidity-sensing applications, but require additional validation (hysteresis, response time, long-term stability) and strict environmental control when employed as stable dielectrics in energy storage or microelectronic devices.

## 5. Conclusions

This work demonstrates that Kramers–Kronig consistency serves as a sensitive diagnostic probe—not merely a binary validity test—for identifying humidity-induced non-idealities or history-dependent processes in nanostructured SiO_2_ MIM capacitors. A reference device measured under ambient conditions exhibited excellent KK consistency for Z′ (NRMSE = 3.3%), confirming that KK-compliant behavior is achievable in these structures. Critically, intra-device humidity characterization on a second device showed progressive KK breakdown: NRMSE for Z′ escalated from 14.5% at 40% RH to 141.2% at 90% RH, directly attributable to moisture-induced ionic conduction and interfacial polarization. Transmission Line Modeling corroborates these findings, revealing a synchronized three-order-of-magnitude reduction in network resistance at high humidity, consistent with water-mediated percolation pathways.

These conclusions are supported by intra-device trends observed under controlled humidity conditions, which provide a methodologically robust basis for interpreting the KK deviations. Due to inherent differences in device geometry and stochastic microstructure, quantitative comparisons between devices are not considered conclusive and are used only for illustrative purposes.

These results demonstrate that humidity-induced KK breakdown in electrosprayed silica nanostructures may be suitable for moisture detection after further metrological validation, while also highlighting that environmental control is essential when such materials are intended as stable dielectrics. Overall, this work offers a rigorous, model-independent diagnostic of environmental effects in nanostructured dielectrics.

## Data Availability

The original contributions presented in this study are included in the article. Raw and processed data are available at [26].

## Supplementary Materials

The following supporting information can be downloaded at: https://www.mdpi.com/article/10.3390/s26102957/s1, Table S1: Fitted parameters of the augmented hybrid Transmission Line Model (TLM) for the nanostructured MIM capacitor under different relative humidity conditions (30%, 50%, 70%, and 90% RH).
