# Supplementary material for: Kramers–Kronig Diagnostic of Humidity-Induced Non-Idealities in Nanostructured Silica Capacitors
Source: Sensors (Basel). 2026 May 8;26(10):2957. doi: 10.3390/s26102957 (PMC13210968; doi:10.3390/s26102957)
Supplement: Supplementary file 1 [file sensors-26-02957-s001.zip › Supplementary Materials.pdf]

Supplementary Materials for

Physical Consistency of Giant Permittivity in Humid Nanostructured Silica Capacitors via Kramers-Kronig Analysis

Bremnen Véliz [1],*, Sendey Vera [1], Sandra Bermejo [2], Albert Orpella [2] and Manuel Domínguez-Pumar [2]

[1] Universidad Estatal Península de Santa Elena, La Libertad, Ecuador; bveliz@upse.edu.ec (B.V.); svera@upse.edu.ec (S.V.)

[2] Universitat Politècnica de Catalunya, Barcelona, Spain; Sandra.bermejo@upc.edu (S.B.); Albert.orpella@upc.edu (A.O.); Manuel.dominguez@upc.edu (M.D.-P.)

Correspondence: bveliz@upse.edu.ec

Journal: Sensors

Section: Physical Sensors

Table S1. Fitted parameters of the augmented hybrid Transmission Line Model (TLM) for the nanostructured MIM capacitor under different relative humidity conditions (30%, 50%, 70%, and 90% RH).

Parameter / Humidity	Units	30% RH	50% RH	70%RH	90% RH
Serial Resistance (R_s)	Ω	~ 0	~ 0	~ 0	~ 0
Total Ionic Resistance (R_i)	Ω	$1.8x10^{+9}$	$4.0x10^{+8}$	$3.9x10^{+7}$	$9.3x10^{+6}$
Total Electronic Resistance (R_e)	Ω	$2.6x10^{+8}$	$3.7x10^{+6}$	$4.5x10^{+6}$	$3.8x10^{+5}$
Ionic Chemical Capacitance ($logC_{\mu i}$)	Log F	-5.1	-4.1	-4.1	-4.9
Electronic Chemical Capacitance ($logC_{\mu e}$)	Log F	-7.0	-7.5	-7.8	-6.1
Dielectric Capacitance ($logC_{diel}$)	Log F	-9.7	-9.8	-9.1	-8.4
Ionic Back Contact Resistance (R_{bi})	Ω	~ 0	~ 0	~ 0	~ 0
Ionic Back Contact Capacitance ($logC_{bi}$)	Log F	N/A	N/A	N/A	N/A
Electronic Back Contact Resistance (R_{be})	Ω	~ 0	~ 0	~ 0	~ 0
Electronic Back Contact Capacitance($logC_{be}$)	Log F	N/A	N/A	N/A	N/A
Ionic Front Contact Resistance (R_{fi})	Ω	$1.9x10^{+6}$	$2.2x10^{+5}$	$1.8x10^{+4}$	$4.3x10^{+3}$
Ionic Front Contact Capacitance($logC_{fi}$)	Log F	-9.5	-9.8	-9.5	-11.0
Leakage Ionic Resistance (R_{li})	Ω	$6.0x10^{+19}$	$3.0x10^{+19}$	$1.4x10^{+11}$	$2.3x10^{+7}$
Electronic Front Contact Resistance (R_{fe})	Ω	~ 0	~ 0	~ 0	~ 0
Electronic Front Contact Capacitance($logC_{fe}$)	Log F	N/A	N/A	N/A	N/A
Inter-rail Interaction Parameter (α_{rail})		0.91	0.74	0.73	0.81
Leakage Electronic Resistance (R_{le})	Ω	$5.1x10^{+4}$	$7.1x10^{+4}$	$7.3x10^{+6}$	$3.2x10^{+7}$

Note: The fitted values for the series and contact resistances (ionic/electronic at the back, and front electronic) are **negligible** (~ 0 Ω). Consequently, these act as short circuits, rendering their parallel associated capacitances **unobservable** (N/A) within the measured frequency range. The physically meaningful trends are observed in the bulk and leakage resistances (R_i, R_e, R_{li}), which show a synchronized three-order-of-magnitude reduction across the humidity range, confirming the enhancement of ionic network connectivity. The inter-rail interaction parameter (α_{ril}) remains stable between 0.73–0.91, indicating consistent coupling despite resistance changes.
